# Comparison of patient reported outcomes after Triathlon^®^ and Kinemax Plus prostheses

**DOI:** 10.1308/003588414X13824511650218

**Published:** 2014-01

**Authors:** S Dixon, AW Blom, MR Whitehouse, V Wylde

**Affiliations:** North Bristol NHS Trust,UK

**Keywords:** Total knee replacement, Patient reported outcomes, Pain, Function, Quality of life, Satisfaction

## Abstract

**INTRODUCTION:**

The Triathlon^®^ (Stryker, Kalamazoo, MI, US) total knee replacement was designed to improve patient function and survivorship. The aim of this study was to determine whether the Triathlon^®^ prosthesis produces better patient reported outcomes than a previous design by the same manufacturer, the Kinemax Plus.

**METHODS:**

The outcome of 233 knees of patients with a mean age of 68 years (range: 40–80 years) who received the Kinemax Plus prosthesis were compared with the outcomes of 220 knees of patients with a mean age of 70 years (range: 42–90 years) who received the Triathlon^®^ prosthesis. Data were collected via postal questionnaire prior to surgery as well as at 8–12 weeks and at 1 year following surgery. Validated questionnaires were used including the WOMAC^®^ (Western Ontario and McMaster Universities) pain and function scales, the Knee injury and Osteoarthritis Outcome Score quality of life scale and the self-administered patient satisfaction scale.

**RESULTS:**

This study found that patients who had the Triathlon^®^ prosthesis had significantly better pain relief (*p*<0.0001), function (*p*=0.028), knee related quality of life (*p*<0.0001) and satisfaction (*p*=0.0003) at three months after surgery than those who received the Kinemax Plus prosthesis. In addition, knee related quality of life (*p*=0.002) and satisfaction (*p*=0.021) were significantly higher at one year after surgery in Triathlon^®^ patients.

**CONCLUSIONS:**

The findings suggest that return to function and reduction in pain may occur more quickly in patients with a Triathlon^®^ prosthesis than in those with the Kinemax Plus.

Total knee replacement (TKR) is one of the most commonly performed elective surgical procedures in the National Health Service (NHS). Projections for the incidence of primary TKR demonstrate potential growth of 673% from 2005 to 2030, with TKR revision burden projected as 7.2% by 2030.^1^ Although estimates are specific to US population data, increased demand for TKR has been demonstrated internationally.^2^ These figures indicate vast economic consequences for healthcare providers.

Historically, outcomes of TKR have been assessed predominantly using surgeon-based measures such as the American Knee Society score^3^ and the Hospital for Special Surgery knee rating score.^4^ More recently, the importance of assessing the outcome of joint replacement from the perspective of the patient has been established.^5,6^ Evidence of the accepted value of investigating outcome from the patient’s perspective is demonstrated with the advent of use of patient reported outcome measures in the NHS as part of a government-led initiative in assessing joint replacement outcomes across England and Wales.^7^ Functional goals following joint replacement can include recreational^6,8^ and sporting pursuits^9^ as well as return to work^10^ in addition to expectations of basic activities of daily living.^11^

New knee prostheses are continuously being developed and introduced to the orthopaedic market. The principles behind the development of the Triathlon^®^ (Stryker, Kalamazoo, MI, US) TKR were to improve patient function and survivorship. The design has an anatomic radius centred on the transepicondylar axis to optimise stability and flexion with flared posterior condyles to allow up to 20º of rotation during flexion. The anterior flange of the femoral component has a 7º angulation to reduce notching with downsized femoral components. The tibial tray incorporates an insert guide with a raised central bar to prevent rotation and an improved locking mechanism. The Triathlon^®^ introduced an annealed, highly cross-linked polyethylene produced with three cycles of gamma irradiation and annealing to optimise wear properties.^12^

Work published in the *Lancet* in 2012 has demonstrated that not all innovation in arthroplasty is an advancement.^13^ We sought to ascertain whether this new design (Triathlon^®^) was an improvement on a previous design by the same manufacturer (Kinemax Plus). The Triathlon^®^ prosthesis has demonstrated better five-year survivorship than the Kinemax Plus, with a revision rate for all causes of 1.56% (95% confidence interval [CI]: 1.2–2.1%) compared with 2.63% (95% CI: 2.3–3.0%).^14^ However, both prostheses demonstrate good survivorship and it has been suggested that if survivorship data are equal, then patient reported outcomes should determine which implant is used.^15^ The aim of this study was therefore to assess patient reported outcomes up to one year after Triathlon^®^ knee replacement and compare them with those of Kinemax Plus patients.

## Methods

Patient reported outcomes from two prospective cohorts (patients with Triathlon^®^ and Kinemax Plus TKR systems) were compared. The Kinemax Plus study, conducted prior to the Triathlon^®^ study, was a prospective multicentre randomised controlled trial involving four hospitals in the UK^16^ while the Triathlon^®^ study, which commenced in 2006, is an ongoing single centre prospective cohort study conducted in England.

Inclusion criteria for both cohorts consisted of patients undergoing primary TKR for osteoarthritis or rheumatoid arthritis. An age limit of 80 years or younger was specified for the Kinemax patients but no age restriction was applied for participation in the Triathlon^®^ study. Exclusion criteria for both studies comprised patients unwilling to provide informed consent, patients with revision surgery or an inability to complete questionnaires for cognitive or physical reasons or language barriers. Additional exclusion criteria for the Kinemax study included history of infection of the knee, instability of the knee preventing the use of an unconstrained prosthesis, and augmentation with wedges and/or structural bone grafting at the time of operation.

Anaesthetic management, operative approach, soft tissue release and resurfacing of the patella were at the discretion of the operating surgeon. All Triathlon^®^ patients received fixed bearing implants (100%) compared with just over half (53%) of the Kinemax group. One hundred and seventy patellas (77%) were resurfaced in the Triathlon^®^ cohort compared with 95 (41%) of the Kinemax cohort. Implants were either cruciate retaining or cruciate sacrificing. These data were not collected for the Kinemax cohort but 202 (92%) of the Triathlon^®^ implants were cruciate retaining. Cementation fixation was employed for all prostheses, using either Palacos^®^ (Heraeus, Wehrheim, Germany) or Simplex™ (Stryker) cement. Twenty-six surgeons participated across both studies and all were consultants or registrars. Postoperative rehabilitation followed the standard practice for each orthopaedic centre. In accordance with the standards set in the Declaration of Helsinki, ethics approval was obtained for both studies and all patients provided informed, written consent.

### Data collection

Data were collected via postal questionnaire prospectively prior to surgery as well as at 8–12 weeks and at 1 year following surgery. Preoperatively, baseline demographic and socioeconomic data were collected, and medical co-morbidities were recorded with the Self-administered Co-morbidity Questionnaire.^17^ For the purposes of assessing knee function, knee pain, knee related quality of life (KRQoL) and satisfaction, the following validated patient reported outcome measures were completed by participants at each assessment time:

*WOMAC^®^ pain and function scales:*^18^ The WOMAC^®^ (Western Ontario and McMasters Universities) questionnaire assesses the severity of pain when performing 5 activities and the degree of functional limitations during 17 activities. The pain and function scores were transformed to a 0–100 scale, with 100 indicating no pain/functional difficulty and 0 indicating extreme pain/functional difficulty.

*Knee injury and Osteoarthritis Outcome Score quality of life scale:*^19^ This 4-item KRQoL scale assesses the extent to which patients are aware of their knee problems and how much they impact on their daily life, with a final score from 0 to 100 (worst to best).

*Self-administered patient satisfaction scale:*^20^ In order to assess satisfaction with the outcome of surgery, a satisfaction questionnaire that has been validated specifically for use in joint replacement patients was included in the postoperative questionnaires. This measure asks about satisfaction with regard to pain relief, ability to perform daily activities, ability to participate in recreational activities and overall satisfaction. Responses are on a four-point scale from very satisfied to very dissatisfied and a global satisfaction scale was calculated (0–100; worst to best).

### Statistical analysis

Multiple regression analysis was performed using InStat^®^ version 3.10 (GraphPad Software, La Jolla, CA, US). Dependent variables were three-month and one-year postoperative scores for WOMAC^®^ pain, WOMAC^®^ function, KRQoL and satisfaction. The independent preoperative variables were the prosthesis type (Triathlon^®^ or Kinemax Plus), patient sex, patient age, diagnosis, body mass index, and preoperative scores for WOMAC^®^ pain, WOMAC^®^ function and KRQoL. The results were checked to see whether multicollinearity was a problem; if the R^2^ value was greater than 0.9, the analysis would be further limited. Where it was not a problem (R^2^<0.75), the analysis was not further modified. Significance was determined with a *p*-value of <0.05.

## Results

Overall, 242 patients (250 knees) received a Kinemax Plus prosthesis and a one-year postoperative questionnaire was completed for 233 knees.^16^ For the Triathlon^®^ study, 251 patients (251 knees) received a Triathlon^®^ prosthesis and a one-year postoperative questionnaire was completed for 220 knees. The mean age of participants who received a Kinemax Plus prosthesis was 68 years (range: 40–80 years, standard deviation [SD]: 7.9 years), which was not significantly different from the mean age of those who received a Triathlon^®^ prosthesis (mean age: 70 years, range: 42–90 years, SD: 9.4 years) (*p*=0.054). A higher percentage of patients who received a Triathlon^®^ prosthesis were female (62%) than those with a Kinemax Plus prosthesis (55%).

### Pain severity

The regression analysis to identify preoperative predictors of postoperative pain severity is displayed in Table 1. Significant predictors of the WOMAC^®^ pain score at three months following surgery were preoperative WOMAC^®^ pain score (*p*=0.021) and the prosthesis type (*p*<0.0001), with the Triathlon^®^ prosthesis demonstrating improved scores (mean: 75, SD: 17.5) compared with the Kinemax Plus prosthesis (mean: 68, SD: 20.7). The only significant predictor of the WOMAC^®^ pain score at one year was the preoperative WOMAC^®^ pain score (*p*=0.004), with lower preoperative pain being associated with lower pain scores at one year.

**Table 1:** Regression analysis to identify preoperative predictors of pain severity at 3 months and 1 year after surgery

	3 months			1 year		
	Regression coefficient	t ratio	*p*-value	Regression coefficient	t ratio	*p*-value
Prosthesis	29.01	1.06	**<0.0001**	3.84	1.91	0.056
Sex	−0.28	0.11	0.914	1.95	1.31	0.192
Age	−0.19	1.23	0.219	0.12	1.36	0.175
Diagnosis	7.26	1.18	0.241	4.65	0.77	0.445
BMI	−0.15	0.61	0.541	0.18	1.72	0.086
WOMAC^®^ pain	0.27	2.32	**0.021**	0.084	2.89	**0.004**
WOMAC^®^ function	−0.94	0.70	0.481	0.099	0.86	0.388
KRQoL	0.020	0.18	0.854	0.079	1.49	0.138

BMI = body mass index; WOMAC^®^ = Western Ontario and McMaster

### Functional ability

The regression analysis to identify preoperative predictors of postoperative functional ability is displayed in Table 2. Significant predictors of the WOMAC^®^ function score at three months following surgery were the preoperative WOMAC^®^ function score (*p*<0.0001) and the prosthesis type (*p*=0.028), with the Triathlon^®^ prosthesis demonstrating improved scores (mean: 71, SD: 17.1) compared with the Kinemax Plus prosthesis (mean: 64, SD: 20.5). The only significant predictor of the WOMAC^®^ function score at one year was the preoperative WOMAC^®^ function score (*p*=0.004), with higher preoperative function being associated with higher function scores at one year.

**Table 2:** Regression analysis to identify preoperative predictors of functional ability at 3 months and 1 year after surgery

	3 months			1 year		
	Regression coefficient	t ratio	*p*-value	Regression coefficient	t ratio	*p*-value
Prosthesis	4.07	2.21	**0.028**	1.13	0.57	0.571
Sex	1.78	0.15	0.883	1.93	1.13	0.260
Age	0.11	1.65	0.101	0.11	1.21	0.226
Diagnosis	4.33	0.59	0.558	4.86	0.98	0.328
BMI	0.16	1.41	0.161	0.18	0.69	0.492
WOMAC^®^ pain	0.08	0.95	0.341	0.08	1.88	0.061
WOMAC^®^ function	0.09	4.40	**<0.0001**	0.10	2.93	**0.004**
KRQoL	0.07	1.07	0.283	0.79	0.25	0.801

BMI = body mass index; WOMAC^®^ = Western Ontario and McMaster

### Knee related quality of life

The regression analysis to identify preoperative predictors of postoperative KRQoL is displayed in Table 3. Younger age (*p*=0.001), a diagnosis of osteoarthritis (*p*=0.029), a better KRQoL score (*p*=0.004) and prosthesis type (*p*<0.0001) (Triathlon^®^ associated with an improved mean score of 57 [SD: 20.8] compared with the mean Kinemax Plus score of 42 [SD: 20.7]) were associated significantly with improved KRQoL scores at three months following surgery. Significant predictors of the KRQoL score at one year were female sex (*p*=0.025), preoperative KRQoL score (*p*=0.021) and prosthesis type (*p*=0.002), with the Triathlon^®^ prosthesis being associated with an improved score (mean: 64, SD: 23.1) compared with the Kinemax Plus prosthesis (mean: 54, SD: 26.4).

**Table 3:** Regression analysis to identify preoperative predictors of knee related quality of life at 3 months and 1 year after surgery

	3 months			1 year		
	Regression coefficient	t ratio	*p*-value	Regression coefficient	t ratio	*p*-value
Prosthesis	13.46	6.48	**<0.0001**	8.01	3.14	**0.002**
Sex	0.34	0.17	0.863	5.56	2.25	**0.025**
Age	0.39	3.28	**0.001**	0.19	1.30	0.196
Diagnosis	10.59	2.19	**>0.029**	0.45	0.08	0.939
BMI	−0.07	0.40	0.690	−0.30	1.32	0.187
WOMAC^®^ pain	−0.02	0.26	0.798	0.18	1.69	0.091
WOMAC^®^ function	0.13	1.31	0.192	−0.09	0.72	0.475
KRQoL	0.24	2.88	**0.004**	0.24	2.31	**0.021**

BMI = body mass index; WOMAC^®^ = Western Ontario and McMaster

### Satisfaction

The regression analysis to identify preoperative predictors of postoperative satisfaction is displayed in Table 4. The only significant predictor of satisfaction at three months and one year following surgery was prosthesis type (*p*=0.0003 and p=0.021 respectively), with the Triathlon^®^ prosthesis being associated with higher satisfaction scores than the Kinemax Plus prosthesis.

**Table 4:** Regression analysis to identify preoperative predictors of satisfaction at three months and one year after surgery

	3 months			1 year		
	Regression coefficient	t ratio	*p*-value	Regression coefficient	t ratio	*p*-value
Prosthesis	9.55	3.70	0.0003	5.51	2.31	0.021
Sex	−0.24	0.10	0.923	2.34	0.07	0.941
Age	0.13	0.89	0.376	0.14	1.45	0.148
Diagnosis	1.93	0.29	0.771	6.27	0.36	0.722
BMI	−0.36	1.60	0.111	0.21	1.84	0.066
WOMAC^®^ pain	0.07	0.70	0.486	0.098	0.77	0.443
WOMAC^®^ function	0.06	0.51	0.609	0.12	1.20	0.233
KRQoL	−0.03	0.27	0.785	0.097	0.76	0.445

BMI = body mass index; WOMAC^®^ = Western Ontario and McMaster Universities; KRQoL = knee related quality of life

## Discussion

The Triathlon^®^ knee system was developed with the aim of providing more natural knee motion and provision of mobility with stability throughout range of movement, and sizing options for Triathlon^®^ have been adapted to allow greater choice for the surgeon and more appropriate sizing for both men and women.^12^ To determine whether the Triathlon^®^ knee replacement produces better outcomes for patients, this study compared patient reported outcomes of the Triathlon^®^ knee replacement with those from an earlier prosthetic design, the Kinemax Plus.

The study found that patients who had a Triathlon^®^ prosthesis had significantly better pain relief, function, KRQoL and satisfaction at three months following surgery than patients who received the Kinemax Plus prosthesis. In addition, KRQoL and satisfaction were significantly higher in patients who had a Triathlon^®^ prosthesis compared with the Kinemax Plus prosthesis at one year. The findings suggest that return to function and reduction in pain may occur more quickly in those with a Triathlon^®^ prosthesis compared with the Kinemax Plus, which could explain satisfaction and KRQoL up to one year following surgery. Expectations are known to strongly influence levels of patient satisfaction.^21^ In demonstrating the Triathlon^®^ prosthesis to be significantly associated with patient satisfaction at three months and one year compared with Kinemax Plus, this may suggest that advances in prosthetic design have been better able to meet the expectations of those patients with Triathlon^®^ TKR.

There has been a limited number of studies investigating outcomes of the Triathlon^®^ knee replacement. In US cohorts of Triathlon^®^ knee replacements, improvements in Knee Society pain and function scores have been high and few complications have been reported.^22,23^ Additionally, participants have demonstrated a rapid return of knee range of motion at six weeks following surgery, with range of motion gradually increasing up to one year.^22^

In a simulator study, Stryker Orthopaedics compared three prosthetic systems (Genesis™ II Oxinium™ [Smith & Nephew, London, UK], conventional Triathlon^®^ and Triathlon^®^ X3^®^) with the aim of investigating the effect of prosthetic design on wear reduction.^24^ Results indicated that both Triathlon^®^ systems demonstrated superior wear resistance to the Genesis™ II Oxinium™ system.

Finally, in 103 Triathlon^®^ TKRs, Cashman *et al* demonstrated no statistically significant differences between groups, measured by WOMAC^®^ and SF-36^®^ (Short Form 36 health survey) at six months, when comparing the use of either intramedullary or extramedullary jigs to align the tibial component.^25^ Consequently, although previous research has found the Triathlon^®^ knee replacement to have resulted in good surgeon-based outcome scores,^22,23^ few complications,^22,23^ low wear rates^24^ and excellent survivorship on the National Joint Registry for England and Wales,^14^ no research has yet assessed the outcomes of the Triathlon^®^ knee replacement from the perspective of the patient.

It cannot be assumed that new prosthetic knee replacements hold advantages over preceding designs in terms of patient outcomes. For that reason, research is paramount in ensuring that the evolution of prosthetic design works in favour of the patient. Clinical and survivorship outcomes provide important information about the implant but they do not necessarily equate with the experiences of the patient.^15^ The inclusion of patient reported outcome measures is essential as they assess the success of the implant from the patient’s perspective, by presenting the realities of joint performance during individual daily demands. We are not aware of other literature investigating patient reported outcomes for the Triathlon^®^ prosthesis or comparisons of Triathlon^®^ with earlier prosthetic designs. This study therefore adds to the literature by demonstrating that the Triathlon^®^ knee replacement produces better patient reported outcomes than the Kinemax Plus prosthesis.

In the regression analyses, several other variables were found to be significant predictors of patient reported outcome at three months and one year following TKR. The only significant predictor of WOMAC^®^ pain and function scores at one year were preoperative WOMAC^®^ pain and function scores, which supports findings from previous research.^26^

Female sex was found to be predictive of higher KRQoL scores at one year, regardless of prosthetic type. This is an unexpected finding as female sex is normally associated with poor baseline and outcome scores of pain and function.^27^ Reports in differences in outcome between sexes have suggested women fail to reach the same level of function as men.^27^ However, an absence of clinically significant sex differences for WOMAC^®^ pain and function from one year following surgery has been demonstrated.^26,28^ Kennedy *et al* suggest similarities exist in rates of recovery following TKR between men and women^29^ although faster recovery in women in the first six months following TKR (measured by WOMAC^®^ pain and function) has been reported more recently.^30^

### Study limitations

Having compared data sets from two different cohorts, the limitations must be acknowledged. First, the Kinemax Plus study was a UK multicentre randomised controlled trial whereas the Triathlon^®^ outcomes study is a single centre cohort study. Results from a multicentre study may be more representative of the TKR population than those from a single centre cohort and this restricts the ability to generalise findings. In the Kinemax trial, fixed and mobile prostheses were compared but no statistical differences were demonstrated between the two designs. Consequently, for the purposes of this investigation, it was possible to treat the data as coming from a single cohort for comparison.

The studies were conducted at different times. Possible adaptations in surgical and TKR management that may influence study outcomes must therefore be considered. Concerning the timeframes in which the Triathlon^®^ and Kinemax Plus and studies were conducted, recent changes in the NHS regarding waiting times for surgery may be reflected in the baseline data. However, differences in baseline status were controlled for in this study using regression analysis. As the Kinemax Plus study is older, there may be differences in participant expectations of surgical outcomes, which could influence reports of satisfaction.

Finally, it is important to consider that although the results of this study demonstrate statistical significance, this does not necessarily imply a meaningful clinical difference between the two prosthetic types. Nevertheless, this study has enabled the comparison of two prosthetic designs, using large sample sizes and a range of validated outcome measures.

## Conclusions

In the short term, patient reported outcomes for the Triathlon^®^ knee replacement may be deemed favourable compared with those of the Kinemax Plus design in terms of pain, function, quality of life and satisfaction. We intend to report longer term outcomes with use of the Triathlon^®^ prosthesis as we continue to follow this cohort prospectively. This will aid in demonstrating whether these outcomes are reflected over longer term use. In order to make sound comparison, future randomised controlled trials are required to confirm the findings of this study.

## Conflict of interest

This research was funded by Stryker UK. The funder had no involvement in the project design, data collection, analysis, interpretation of data or writing of the manuscript.
